# EMT Markers in Locally-Advanced Prostate Cancer: Predicting Recurrence?

**DOI:** 10.3389/fonc.2019.00131

**Published:** 2019-03-11

**Authors:** Katia A. Cheaito, Hisham F. Bahmad, Ola Hadadeh, Eman Saleh, Christelle Dagher, Miza Salim Hammoud, Mohammad Shahait, Zaki Abou Mrad, Samer Nassif, Ayman Tawil, Muhammad Bulbul, Raja Khauli, Wassim Wazzan, Rami Nasr, Ali Shamseddine, Sally Temraz, Marwan E. El-Sabban, Albert El-Hajj, Deborah Mukherji, Wassim Abou-Kheir

**Affiliations:** ^1^Department of Anatomy, Cell Biology and Physiological Sciences, Faculty of Medicine, American University of Beirut, Beirut, Lebanon; ^2^Division of Hematology/Oncology, Department of Internal Medicine, American University of Beirut Medical Center, Beirut, Lebanon; ^3^Division of Urology, Department of Surgery, American University of Beirut Medical Center, Beirut, Lebanon; ^4^Department of Pathology and Laboratory Medicine, American University of Beirut Medical Center, Beirut, Lebanon

**Keywords:** prostate cancer, cytokeratin 8, vimentin, epithelial-to-mesenchymal transition, Gleason group, clinicopathological parameters

## Abstract

**Background:** Prostate cancer (PCa) is the second most frequent cause of cancer-related death in men worldwide. It is a heterogeneous disease at molecular and clinical levels which makes its prognosis and treatment outcome hard to predict. The epithelial-to-mesenchymal transition (EMT) marks a key step in the invasion and malignant progression of PCa. We sought to assess the co-expression of epithelial cytokeratin 8 (CK8) and mesenchymal vimentin (Vim) in locally-advanced PCa as indicators of EMT and consequently predictors of the progression status of the disease.

**Methods:** Co-expression of CK8 and Vim was evaluated by immunofluorescence (IF) on paraffin-embedded tissue sections of 122 patients with PCa who underwent radical prostatectomies between 1998 and 2016 at the American University of Beirut Medical Center (AUBMC). EMT score was calculated accordingly and then correlated with the patients' clinicopathological parameters and PSA failure.

**Results:** The co-expression of CK8/Vim (EMT score), was associated with increasing Gleason group. A highly significant linear association was detected wherein higher Gleason group was associated with higher mean EMT score. In addition, the median estimated biochemical recurrence-free survival for patients with < 25% EMT score was almost double that of patients with more than 25%. The validity of this score for prediction of prognosis was further demonstrated using cox regression model. Our data also confirmed that the EMT score can predict PSA failure irrespective of Gleason group, pathological stage, or surgical margins.

**Conclusion:** This study suggests that assessment of molecular markers of EMT, particularly CK8 and Vim, in radical prostatectomy specimens, in addition to conventional clinicopathological prognostic parameters, can aid in the development of a novel system for predicting the prognosis of locally-advanced PCa.

## Introduction

Prostate Cancer (PCa) is the second most frequently diagnosed cancer and the sixth leading cause of cancer death in males worldwide (1). Screening for PCa is not routinely practiced in the Middle East, which pertains to the rising incidence rates and the high proportion of patients being diagnosed with high-risk locally-advanced and metastatic disease in this region of the world (2, 3).

Radical prostatectomy is an effective therapeutic procedure for men with organ-confined PCa. This modality, however, fails in 30–40% of patients as serum prostate-specific antigen (PSA) levels continue to rise and patients eventually develop biochemical recurrence postoperatively (4). It is of utmost importance to identify the parameters that can accurately predict the prognosis and clinical outcome following radical prostatectomy. To date, several investigators have described the usefulness of various clinicopathological factors—including PSA, Gleason scores, pathological stage, surgical margin status (SMS), perineural invasion (PNI), seminal vesicle invasion (SVI), lymphovascular invasion (LVI), and tumor volume—and their correlation with treatment failure (5–8). However, these studies have carried several limitations, such as the recent stage migration and grade inflation because of the greater aggressiveness of PCa (9), besides the differences in PCa features among diverse ethnic groups (10).

Expression of epithelial-to-mesenchymal transition (EMT) markers represents a crucial step in the malignant progression of several cancers, such as prostate, breast, ovarian, and colon cancers (11–15). This pathological process ensues the breakdown of cell-to-cell or cell-to-extracellular matrix (ECM) adhesions at the polarized epithelium lining prompting conversion into mesenchymal phenotype and enhanced cell mobility, invasion, and metastasis (14, 16). The role of EMT in PCa metastasis has been studied (16) revealing significant interplay between EMT-related genes and tissue invasion on one hand, and alterations in TGF-β (17), IL-6 (18–20), AR variants (21, 22), FGF (23), and Wnt/β-catenin signaling pathways (24–26) on the other hand.

In a previous study by our group, we have reported increased co-expression of epithelial cytokeratin 8 (CK8) and mesenchymal vimentin (Vim) markers in androgen-independent PLum-AI murine PCa cell lines, which represent advanced stages of PCa, referring to a positive EMT status in those cells, when compared to androgen-dependent PLum-AD cells which represent primary PCa (27). CK8/Vim co-expression was also reported in other murine PCa cell lines, including PLum-P and PLum-C *Pten*^−/−^ *TP53*^−/−^ murine prostate epithelial progenitor cells (28).

In this study, we evaluated the co-expression of two potential molecular markers of EMT, namely CK8 and Vim, in radical prostatectomy specimens of locally-advanced PCa patients using immunofluorescent (IF) staining. Accordingly, we developed a novel scoring system to quantify EMT expression (EMT score) and explored the correlation between this score and the different clinicopathological outcomes. Our results confirmed that the EMT score can predict PSA failure, and thus biochemical recurrence, irrespective of Gleason group and other conventional PCa diagnostic and prognostic parameters.

## Materials and Methods

### Patients Selection

Using the radical prostatectomy institutional database (1998–2016) of the American University of Beirut Medical Center (AUBMC), we identified 122 patients with locally-advanced PCa. Those patients had adverse pathological features with more than 30 months of follow-up. The study with all its experimental protocols was conducted under the Institutional Review Board (IRB) approvals of the American University of Beirut (AUB) and AUBMC. The work described herein has been carried out in accordance with relevant guidelines and regulations, and in agreement with The Code of Ethics of the World Medical Association (Declaration of Helsinki) for experiments involving human subjects.

### Clinicopathological Variables

Preoperative serum PSA level, Gleason group, pathological stage, positive surgical margin (PSM), perineural invasion (PNI), seminal vesicle invasion (SVI), lymphovascular invasion (LVI), and tumor volume in the PCa specimens were recorded.

### Tissue Sampling and Gleason Scoring and Grouping

**Figure 1:**
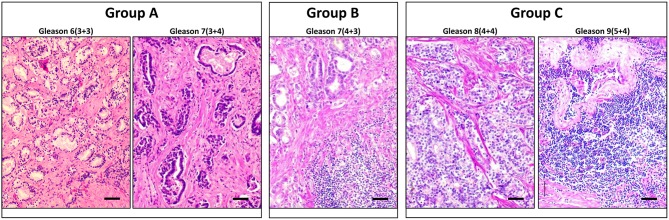
Representative H and E staining of PCa tissue sections that represent each of the three Gleason groups. Cross-sections of PCa tissues representing each of the three Gleason groups are stained with H and E. **Left panel** represents group A that includes Gleason scores 6 and 7(3+4). **Middle panel** represents Group B that includes Gleason score 7(4+3). **Right panel** represents group C that includes Gleason scores 8 and 9. Scale bars = 50 μm.

The tumor tissues were harvested and fixed in 4% formalin overnight, rinsed well in PBS and transferred to 70% ethanol before standard processing to obtain paraffin-embedded sections. Blocks of tumor tissues were identified by pathologists in terms of quality and content, and slides with unstained sections were obtained along with the H&E sections as a reference. The tumor grade and clinical stage were reviewed, and the Gleason scores were assigned by two independent pathologists according to the International Society of Urological Pathology (ISUP) criteria. This new five–grade group system has been suggested by the ISUP and accepted by the WHO in 2016, in order to address the deficiencies in the previous Gleason scoring systems (29). The sections were immunostained and analyzed for CK8/Vim co-expression, and the EMT score was then compared between three different Gleason groups that we assigned: group A (grade groups 1 and 2); group B (grade group 3); and group C (grade groups 4 and 5). Representative images of the H&E staining of PCa tissue sections that represent each of the three Gleason groups are shown in Figure 1.

### Antibodies and Reagents

Antibodies used in this study include mouse monoclonal anti-CK8 (1/200 dilution) (Covance, CA), rabbit polyclonal anti-Vim (1/50 dilution) (Santa Cruz Biotechnology, CA), Alexa 488 goat anti-rabbit, and Alexa 568 goat anti-mouse (Invitrogen, CA). All secondary Alexa Fluor antibodies were used at 1/200 dilution. Fluoro-gel II with DAPI (Electron Microscopy Sciences, PA) was used for mounting.

### Immunofluorescent Staining Procedure for Tissues

Unstained formalin-fixed paraffin-embedded (FFPE) tissue sections were deparaffinized, and antigen retrieval was performed in a citrate buffer in a steamer at 100°C for 40 min. This was followed by protein blocking using the blocking buffer (3% BSA, 0.1% Triton x-100, and 10% Normal Goat Serum in PBS) for an hour at room temperature. Slides were stained using the different primary antibodies: anti-CK8 overnight, and anti-Vim for 2 h; then tissues were incubated with the corresponding secondary antibodies. Finally, slides were mounted with the anti-fade Fluoro-gel II with DAPI.

### Microscope Specifications

Indirect immunofluorescence microscopic analyses were performed using Carl Zeiss Axio Observer.Z1 and LSM710 laser scanning confocal microscopes. All images were acquired and analyzed using the Carl Zeiss ZEN 2012 image software.

### IF Evaluation and EMT Scoring

EMT scoring was performed manually using a 40 × objective and a Carl Zeiss Axio Observer.Z1 microscope. It was done by screening the whole tissue section in a systematic manner and counting the total number of glands, then counting the number of glands with at least one cell co-expressing CK8 and Vim. Then, the percentage was calculated by dividing the number of glands with at least one double positive cell by total number of glands, multiplied by 100. This percentage is referred to as EMT score. CK8/Vim staining was graded as double positive only when cytoplasmic staining was detectable.

### Statistical Analysis

The EMT score was categorized into < 25% and more than or equal to 25%. This cutoff of 25% was assigned based on the EMT score distribution where 95.1% (116) of the total population clustered in the “less than or equal to 50% EMT score.” Chi-square test and two-tailed unpaired Student's *t*-test of independent variables were used to assess the association of the EMT score categorized into two groups with the sample clinicopathological characteristics such as age, PCa pathological stage, preoperative PSA, PSA failure, percentage of tumor volume involved, prostate size, perineural invasion, SVI, lympho-vascular invasion, and surgical margins. Student *t*-test of independent variables was used to compare the mean EMT score (as a continuous variable) between the different Gleason groups. A Mantel–Haenszel test of trend was run to determine whether a linear association existed between the EMT score categories and the different Gleason groups. In a secondary analysis, a linear regression model was built to examine the effect of the Gleason group on the EMT score while adjusting for the pathological stage and the surgical margins. EMT score in addition to the Gleason group, pathological stage, and surgical margins (the three clinicopathological variables which showed statistically significant difference between the two EMT score categories) were entered as covariates in the cox regression model. *P* ≤ 0.05 were considered significant. Statistical analysis was performed using the Statistical Package for the Social Sciences statistical package 21.0 software (SPSS, Inc.).

## Results

### Clinicopathological Characteristics of PCa Patients and Their Correlation With the EMT Score

**Figure 2:**
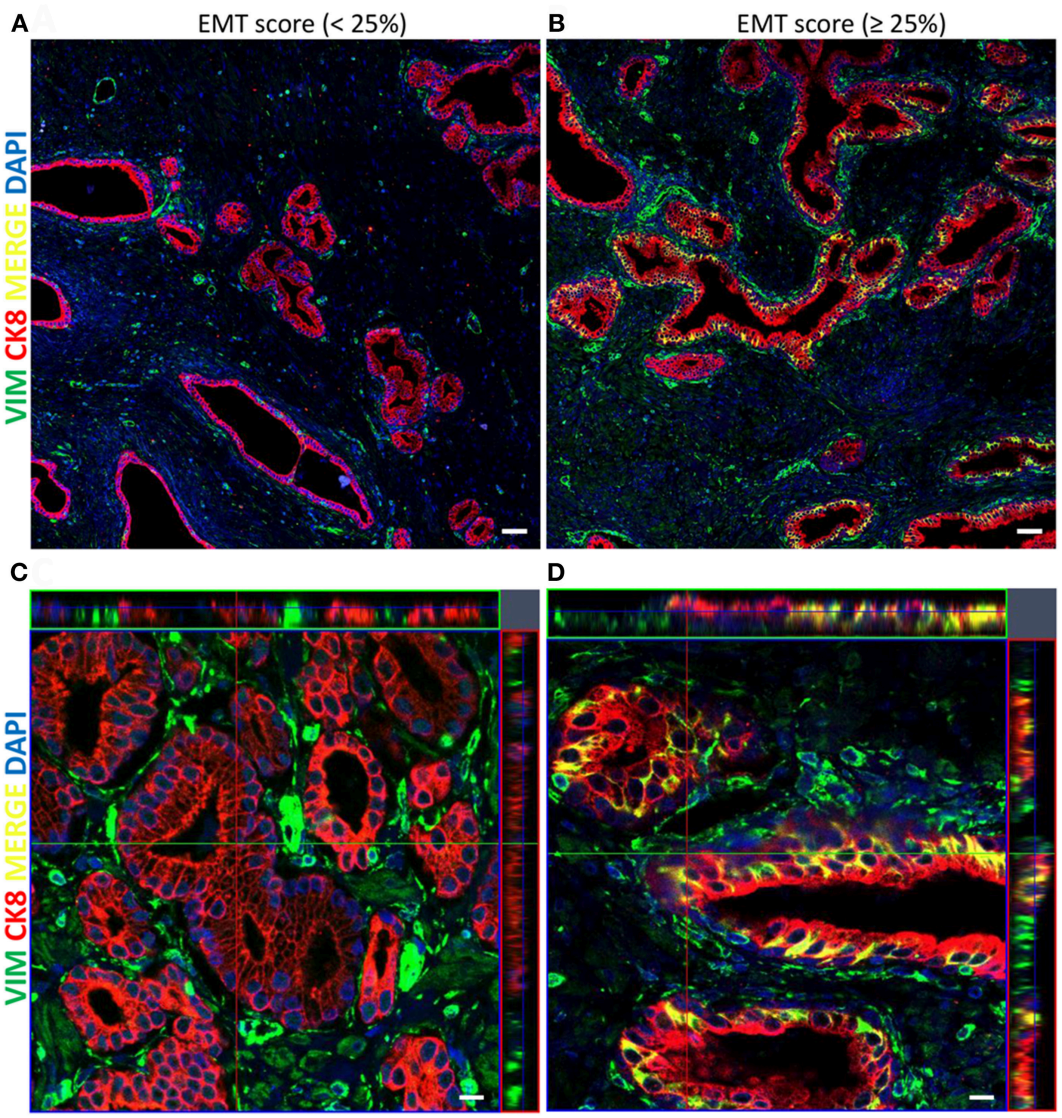
Representative immunofluorescent images of the co-expression of CK8/Vim molecular markers in PCa tissue specimens stained with CK8 (red), Vimentin (green), and DAPI (blue). **(A)** Tile scan image (5 x 5) of PCa tissue showing low EMT score <25% (scale bar = 50μm). **(B)** Tile scan image (5 x 5) of PCa tissue showing high EMT score ≥25% (scale bar = 50 μm). **(C)** Z-stack with maximal and orthogonal projection of PCa tissue showing low EMT score <25% (scale bar = 10 μm). **(D)** Z-stack with maximal and orthogonal projection of PCa tissue showing high EMT score ≥25% (scale bar = 10μm).

A total of 122 radical prostatectomy specimens were analyzed. Table 1 summarizes the clinicopathological characteristics of the 122 patients. Association of several clinicopathological variables and EMT score is shown in Table 2. The specimens were analyzed by IF and examined for CK8/Vim co-expression (EMT score) (Figure 2).

**Table 1:** Clinicopathological characteristics of the 122 patients with PCa included in our study.

**Clinicopathological variable**	**Total *N***	**Categories**	***n* (%)**
Age (in years)	122	Mean (±SD)	62.1(±6.5)
		< 70	109 (89.3)
		≥70	13 (10.7)
Gleason groups	122	A: Gleason scores 6 and 7(3 + 4)	63 (51.6)
		B: Gleason score 7(4 + 3)	30 (24.6)
		C: Gleason scores 8 and 9	29 (23.8)
Lympho-vascular invasion	61	Absent	55 (90.2)
		Present	6 (9.8)
Perineural invasion	94	Absent	28 (29.8)
		Present	66 (70.2)
Seminal vesicle invasion	118	Absent	96 (81.4)
		Present	22 (18.6)
Lymph node invasion	28	Absent	25 (89.3)
		Present	3 (10.7)
Pathological stage	120	pT2	35 (29.2)
		≥pT3	85 (70.8)
Preoperative PSA (in ng/mL)	116	Mean (±SD)	10.7(±9.8)
		< 10	76 (65.5)
		≥10	40 (34.5)
Prostate size (in g)	120	Mean (±SD)	58.3 (±59.5)
		< 50	65 (54.2)
		≥50	55 (45.8)
Tumor volume (in cc)	113	Mean (±SD)	14.9 (±21.9)
		< 5	26 (23)
		≥5	87 (77)
PSA failure	87	No	45 (51.7)
		Yes	42 (48.3)

**Table 2:** Correlation of EMT score with the patients' clinicopathological variables.

**Clinicopathological variable**		**EMT score**			***P*-value**
		**<25**	**≥25**	**Total**	
		***N* (%)**	***N* (%)**	***N* (%)**	
Age (in years)	Mean (±SD)	61.6 (±6.2)	63.6 (±7.2)	62 (±6.5)	0.167
	<70	85 (93.4)	22 (81.5)	107 (90.7)	0.061
	≥70	6 (6.6)	5 (18.5)	11 (9.3)	
	Total	91 (100)	27 (100)	118 (100)	
Gleason groups	A: Gleason scores 6 and 7(3 + 4)	51 (56)	9 (33.3)	60 (50.8)	**0.014**
	B: Gleason score 7(4 + 3)	24 (26.4)	6 (22.2)	30 (25.4)	
	C: Gleason scores 8 and 9	16 (17.6)	12 (44.4)	28 (23.7)	
	Total	91 (100)	27 (100)	118 (100)	
Lympho-vascular invasion	Absent	32 (88.9)	22 (91.7)	54 (90)	0.725
	Present	4 (11.1)	2 (8.3)	6 (10)	
	Total	36 (100)	24 (100)	60 (100)	
Perineural invasion	Absent	15 (23.1)	10 (38.5)	25 (27.5)	0.137
	Present	50 (76.9)	16 (61.5)	66 (72.5)	
	Total	65 (100)	26 (100)	91 (100)	
Seminal vesicle invasion	Absent	74 (83.1)	19 (73.1)	93 (80.9)	0.251
	Present	15 (16.9)	7 (26.9)	22 (19.1)	
	Total	89 (100)	26 (100)	115 (100)	
Lymph node invasion	Absent	11 (91.7)	13 (86.7)	24 (88.9)	0.681
	Present	1 (8.3)	2 (13.3)	3 (11.1)	
	Total	12 (100)	15 (100)	27 (100)	
Pathological stage	pT2	21 (23.6)	13 (48.1)	34 (29.3)	**0.014**
	≥pT3	68 (76.4)	14 (51.9)	82 (70.7)	
	Total	89 (100)	27 (100)	116 (100)	
PSA failure	No	32 (49.2)	11 (55)	43 (50.6)	0.625
	Yes	33 (50.8)	9 (45)	42 (49.4)	
	Total	65 (100)	20 (100)	85 (100)	
Surgical margins	Negative	17 (18.7)	12 (44.4)	29 (24.6)	**0.006**
	Positive	74 (81.3)	15 (55.6)	89 (75.4)	
	Total	91 (100)	27 (100)	118 (100)	
Preoperative PSA (in ng/mL)	Mean (±SD)	10 (±7.1)	13.7 (±16.4)	10.9 (±9.9)	0.289
	< 10	55 (63.2)	17 (68)	72 (64.3)	0.66
	≥10	32 (36.8)	8 (32)	40 (35.7)	
	Total	87 (100)	25 (100)	112 (100)	
Prostate size (in g)	Mean (±SD)	58.5 (±67.7)	60.1 (±24.8)	58.9 (±60.4)	0.903
	< 50	48 (53.9)	13 (48.1)	61 (52.6)	0.598
	≥50	41 (46.1)	14 (51.9)	55 (47.4)	
	Total	89 (100)	27 (100)	116 (100)	
Tumor volume (in cc)	Mean (±SD)	16.1 (±24.7)	12.3 (±9.4)	15.2 (±22.2)	0.461
	< 5	18 (21.4)	7 (28)	25 (22.9)	0.493
	≥5	66 (78.6)	18 (72)	84 (77.1)	
	Total	84 (100)	25 (100)	109 (100)	

*Bold values represent statistically significant data*.

In studying the sample distribution statistics between the two categories of the EMT score, a significant statistical difference was detected between the two categories in terms of Gleason group (*p* = 0.014), pathological stage (*p* = 0.014), and surgical margins (*p* = 0.006). No significant differences in the patient's age, pre-operative PSA, PSA failure (defined by an increase in blood PSA level at or above 0.2 ng/mL following surgery), and tumor volume were observed (Table 2).

### High Mean EMT Score Is Significantly Associated With Higher Gleason Group

To investigate the difference in the mean EMT score between the assigned Gleason groups, an independent *t*-test was run. There were 60 patients in group A, 30 patients in group B, and 28 patients in group C. There was no statistical difference in the mean EMT score between group A and B. Nonetheless, the mean EMT score was higher in group B (*M* = 15.3%, *SD* = 21.3%) than group A (*M* = 10.7 %, *SD* = 11.6%), with a mean difference (*M* = −4.62, 95% CI [−13.04;3.8], *p* = 0.274). When comparing the mean EMT score of the 60 patients in the Gleason group A (*M* = 10.7 %, *SD* = 11.6%) to the 28 patients in group C (*M* = 26.8%, *SD* = 29.1%), a significant difference with quite high mean difference was recorded (*M* = −16.09, 95% CI [−27.71; −4.47], *p* = 0.008). The mean EMT score comparison between groups B and C revealed no significant difference, although a higher mean was recorded in the higher Gleason group (*M* = −11.47, 95% CI [−24.99; −2.06], *p* = 0.091) (Table 3).

**Table 3:** Comparison of the mean EMT scores between Gleason groups.

	**Gleason group**	***N***	**Mean (±*SD*)**	**Mean difference (95% CI)**	***P*-value**
Mean EMT score	A: Gleason scores 6 and 7(3 + 4)	60	10.7 (±11.6)	−4.62 [−13.04; 3.8]	0.274
	B: Gleason score 7(4 + 3)	30	15.3 (±21.3)		
	B: Gleason score 7(4 + 3)	30	15.3 (±21.3)	−11.47 [−24.99; 2.06]	0.091
	C: Gleason scores 8 and 9	28	26.8 (±29.1)		
	A: Gleason scores 6 and 7(3 + 4)	60	10.7 (±11.6)	−16.09 [−27.71; −4.47]	**0.008**
	C: Gleason scores 8 and 9	28	26.8 (±29.1)		

*Bold values represent statistically significant data*.

A mean plot of the EMT score vs. the three Gleason groups is shown in Supplementary Figure 1. A Mantel–Haenszel test of trend was run to determine whether a linear association existed between EMT score categorized into two groups (< 25% and more than or equal to 25%) and the assigned Gleason groups. The Mantel–Haenszel test of trend showed a statistically significant linear association between them [$\chi_{\left(1\right)}^{2}$ = 7.547, *p* < 0.007, *r* = 0.254], where higher Gleason group was associated with a higher EMT score (Supplementary Table 1). A scatterplot simplifying the linear association between EMT score and the Gleason groups is presented in Supplementary Figure 2.

### Gleason Groups Can Predict EMT Score Irrespective of the Pathological Stage and Surgical Margins

A multiple regression model was built to study if Gleason group can predict EMT score while adjusting for the pathological stage and surgical margins, the variables which showed statistically significant difference between the two EMT score categories (Table 2). The multiple regression model significantly predicted EMT score, *F*_(3, 112)_ = 7.037, *p* < 0.001. *R*^2^ for the overall model was 15.9 % with an adjusted *R*^2^ of 13.6%. Only Gleason group added statistical significance to the prediction, *p* = 0.001. Regression coefficients and their *P*-values can be found in Supplementary Table 2.

### EMT Score Can Predict PSA Failure Irrespective of Gleason Group, Pathological Stage, or Surgical Margins

**Figure 3:**
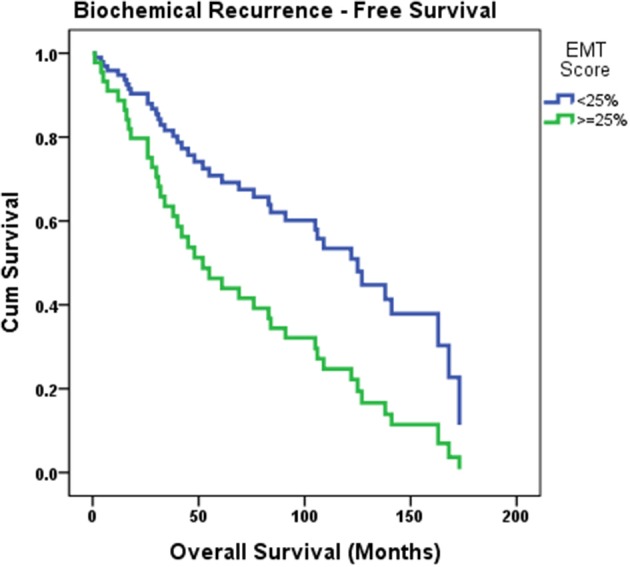
Biochemical recurrence-free survival curve estimating PSA failure based on the patients' EMT score. Cox regression model was built where time to PSA failure was considered time to event, and EMT score, Gleason group, pathological stage and surgical margins were added as covariates to the model. Biochemical recurrence was found to be higher in patients with EMT score ≥25% (*p* = 0.045).

To study the correlation between EMT score and PSA failure, a Cox regression model was built. Time to PSA failure was considered time to event, and EMT score, Gleason group, pathological stage, and surgical margins were added as covariates to the model using forward method. EMT score was found to be an independent predictor of PSA failure. Biochemical recurrence was higher in patients with EMT score ≥25% (OR: 2.23, 95% CI [1.018; 4.895], *p* = 0.045). The overall model has a χ^2^ of 4.221, with a *P*-value of 0.04. Biochemical recurrence-free survival curve estimating PSA failure based on the patients' EMT score is shown in Figure 3.

## Discussion

Despite the advances in the treatment of metastatic PCa, most patients eventually die from their disease. This is due to the rapid and poorly understood progression of PCa from a primary stage to an advanced and metastatic castration-resistant PCa (mCRPC) stage which involves several mechanisms, including epithelial-to-mesenchymal transition (EMT). The latter is recognized in endorsing the invasiveness of PCa cells due to increased mobility and migration of mesenchymal cells (16). In addition to the role of EMT in PCa progression, it has been identified as playing a substantial role in PCa therapeutic resistance to anti-androgens and radiotherapy (30). Therefore, it has been postulated that targeting EMT may improve the overall survival of patients with PCa (16). The main cause of PCa mortality is the progression to metastatic castration-resistant PCa (mCRPC); therefore, identifying the onset of metastatic dissemination through assessment of molecular markers of EMT can aid in the development of a novel system for predicting the prognosis of PCa. Nonetheless, the translation of EMT into clinical applicability presents substantial challenges (31). This can be attributed to tumor heterogeneity and diverse metastatic behavior, which is underrepresented in currently used homogenous cell lines and preclinical models (32). Yet, several studies have addressed the changes in the expression levels of genes and/or proteins associated with EMT in human tumor samples to establish an association with clinical significance.

In carcinoma, invasion and metastasis are associated with transition of cancer cells form an epithelial keratins-expressing phenotype to a mesenchymal vimentin (Vim)-expressing phenotype (33, 34). The importance of assessing the EMT status through investigating Vim overexpression was highlighted in different solid malignancies. For instance, Vim expression was associated with adverse prognosis in ductal breast carcinoma (35). Besides, in triple-negative breast cancer, Vim expression was significantly higher compared to other subtypes, and was shown to be associated with a worse prognosis and a more aggressive phenotype, thereby assisting as a biomarker for the prognosis of this aggressive subtype of breast cancer (36). In a study by Bukhari et al., Vim was also suggested to aid in predicting the risk of developing colon cancer and its use was proposed to serve as an antigen for tumor vaccination for colon cancers (37). Additionally, a significant increase in Vim expression coupled with a decrease in cytokeratin expression were observed in advanced grades of transitional cell carcinoma of the bladder, suggesting the potential use of these biomarkers for early diagnosis of bladder carcinoma (38). In PCa, Gravdal et al. focused on the independent relationship between an E-cadherin to N-cadherin switch and patient prognosis by unraveling the importance of EMT in PCa progression (39).

In the present study, we investigated the correlation between EMT score on one hand, designated by estimating the percentage of glands co-expressing epithelial CK8 and mesenchymal Vim markers out of the total glands counted within a PCa radical prostatectomy tissue section, and the various clinicopathological parameters among locally-advanced PCa patients on the other hand. The value of Vim expression as a predictor of recurrence was established in a previous study where Zhang et al. performed an immunohistochemical study and reported that risk of biochemical recurrence is associated with high levels of Vim which was described to be independent of Gleason score (40). In our patients, representing a cohort of high-risk locally-advanced PCa from the Middle East region, looking at co-expression patterns of CK8 and Vim revealed that the mean EMT score increases significantly as disease becomes more poorly differentiated reflected by higher Gleason group (Table 3). Our results show that there is a highly significant difference in the mean EMT score between Gleason groups A and C (10.7 ± 11.6% in Gleason group A vs. 26.8 ± 29.1% in Gleason group C, *p* = 0.008). Furthermore, there is a highly significant linear association based on Mantel–Haenszel test (*p* = 0.007) whereby higher Gleason groups were associated with higher EMT scores (Supplementary Figure 2). The added value of this EMT scoring system is the fact that it can predict PSA failure irrespective of Gleason group, pathological stage, and surgical margins (41). As PSA recurrence is a powerful predictor of distant metastasis, cancer-specific survival, and overall survival, these results suggest that the EMT score can be used to estimate the biochemical recurrence-free survival of a patient irrespective of other clinicopathological parameters.

A possible explanation of the link between EMT status and disease progression is the fact that cells with hybrid epithelial/mesenchymal phenotypes possess a large repertoire of survival strategies under many stress conditions (42). EMT has been linked to circulating tumor cells (CTCs) generation and subsequently metastasis. In colorectal cancer, for instance, the presence of biophenotypic and mesenchymal CTCs, rather than epithelial CTCs, is indicative of a more advanced disease stage and metastasis (43).

## Conclusions

In conclusion, this study underscores the importance of EMT markers (increased Vim and decreased CK8 expression) for predicting the prognosis of PCa. Whereas, previous studies have indicated reduced expression of epithelial markers and increasing expression of mesenchymal markers, an EMT phenotype and the co-expression of such markers specifically CK8 and Vim and their association with outcome data have not been described. Since these markers could have a significant effect on the management of PCa patients, including projections of targeted therapy, we suggest the extrapolation of this study to larger cohorts of patients from different ethnicities to further validate our findings. Besides, since androgen receptor (AR) expression and EMT have been recently reported to be mutually exclusive (44), future studies are indeed warranted to evaluate expression levels of AR and PSA in the PCa tissue samples and their correlation with EMT score.

## Study Limitations

We recognize that our study has some limitations. First, as a clinical study the sample size is relatively small, therefore the results obtained require further investigation on a larger cohort. Second, samples were collected retrospectively over the period of 18 years with around 75% of the samples having a positive margin and around 70% with a pathological stage greater than pT3. The latter identified the study sample as a high-risk cohort thus restricting the results obtained to such sample characteristics. Third, the retrospective collection of data led to missing information regarding the SVI, PNI, and LNM status of the patients; this might explain the lack of significant correlation between the EMT score and the metastatic status.

## Author Contributions

KC, HB, OH, ES, CD, MH, MS, ZM, SN, AT, MB, RK, WW, RN, AS, ST, and ME-S contributed to the project design and execution of experiments. KC, HB, OH, and ES contributed to the analysis of results and writing of manuscript. AE-H, DM, and WA-K contributed to overlooking and following up with experiments, result analysis, and manuscript proofreading. AE-H, DM, and WA-K contributed to project design, result analysis, manuscript writing, and proofreading. All authors critically revised and edited the manuscript and approved the final draft.

### Conflict of Interest Statement

The authors declare that the research was conducted in the absence of any commercial or financial relationships that could be construed as a potential conflict of interest.

## Supplementary Material

Supplementary Table 1 Gleason group distribution among the EMT score categories.

Supplementary Table 2 Regression coefficients of the multiple regression model.

Supplementary Figure 1 Mean plot of mean EMT score in percentage vs. different Gleason groups showing a linear association. Linear association exists between mean EMT score and the assigned Gleason groups A; Gleason scores 6 and 7(3 + 4), group B; Gleason score 7(4 + 3), and group C; Gleason scores 8 and 9, where the mean percentage EMT score increases drastically when the gleason group increases.

Supplementary Figure 2 Scatterplot of the EMT score vs. different Gleason groups showing a linear association. A Mantel–Haenszel test of trend was run to determine whether a linear association existed between EMT score categorized into two groups (<25 and ≥25%) and the assigned Gleason groups revealing a statistically significant linear association between them (Supplementary Table 1, *p* < 0.007), where higher Gleason group was associated with a higher EMT score.
