# Supplementary material for: EMT Markers in Locally-Advanced Prostate Cancer: Predicting Recurrence?
Source: Front Oncol. 2019 Mar 11;9:131. doi: 10.3389/fonc.2019.00131 (PMC6421270; doi:10.3389/fonc.2019.00131)
Supplement: Supplementary Table 1 — Gleason group distribution among the EMT score categories. [file Table_1.docx]

**Supplementary Table 1.** Gleason group distribution among the EMT score categories.

| Gleason Group | EMT Score | | |
| --- | --- | --- | --- |
|  | **< 25** | **≥ 25** | **Total** |
| A: Gleason scores 6 and 7(3+4) | 51 (56%) | 9 (33.3%) | 60 (50.8%) |
| B: Gleason score 7(4+3) | 24 (26.4%) | 6 (22.2%) | 30 (25.4%) |
| C: Gleason scores 8 and 9 | 16 (17.6%) | 12 (44.4%) | 28 (23.7%) |
| Total | 91 (100%) | 27 (100%) | 118 (100%) |
