# Supplementary material for: EMT Markers in Locally-Advanced Prostate Cancer: Predicting Recurrence?
Source: Front Oncol. 2019 Mar 11;9:131. doi: 10.3389/fonc.2019.00131 (PMC6421270; doi:10.3389/fonc.2019.00131)
Supplement: Supplementary Table 2 — Regression coefficients of the multiple regression model. [file Table_2.docx]

**Supplementary Table 2.** Regression coefficients of the multiple regression model.

| Clinicopathological variable | B (95% CI) | P-value |
| --- | --- | --- |
| Gleason group | 7.38 [8.2;45.63] | **0.001** |
| Pathological stage | -6.79 [3.06;11.71] | 0.118 |
| Surgical margins | -7.08 [-15.32;1.74] | 0.122 |
