# Supplementary figures and images for: EMT Markers in Locally-Advanced Prostate Cancer: Predicting Recurrence?
Source: Front Oncol. 2019 Mar 11;9:131. doi: 10.3389/fonc.2019.00131 (PMC6421270; doi:10.3389/fonc.2019.00131)

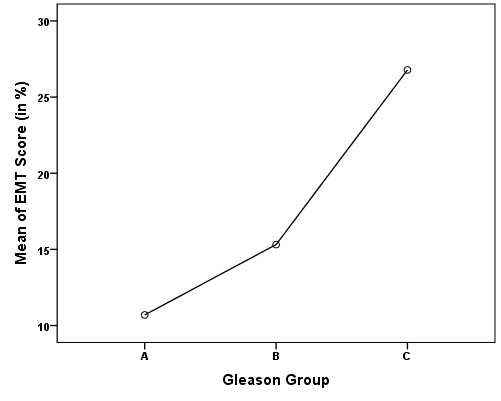

Supplement: Supplementary Figure 1 — Mean plot of mean EMT score in percentage vs. different Gleason groups showing a linear association. Linear association exists between mean EMT score and the assigned Gleason groups A; Gleason scores 6 and 7(3 + 4), group B; Gleason score 7(4 + 3), and group C; Gleason scores 8 and 9, where the mean percentage EMT score increases drastically when the gleason group increases. [file Image_1.TIF]

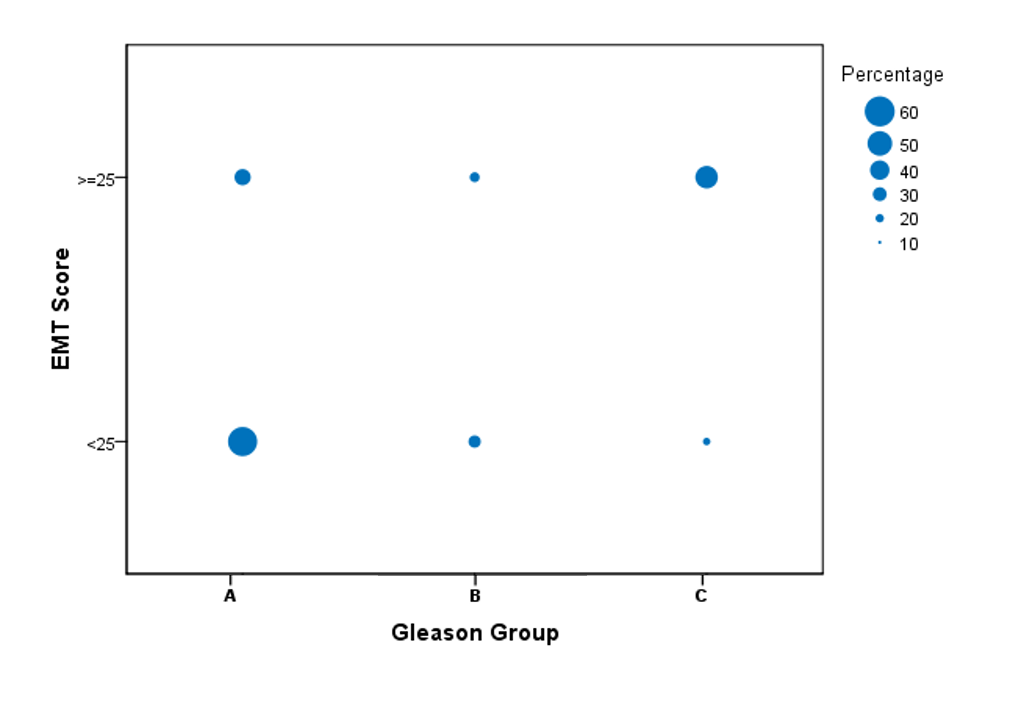

Supplement: Supplementary Figure 2 — Scatterplot of the EMT score vs. different Gleason groups showing a linear association. A Mantel–Haenszel test of trend was run to determine whether a linear association existed between EMT score categorized into two groups (<25 and ≥25%) and the assigned Gleason groups revealing a statistically significant linear association between them (Supplementary Table 1, p < 0.007), where higher Gleason group was associated with a higher EMT score. [file Image_2.TIF]
